# A Detection Method of Operated Fake-Images Using Robust Hashing

**DOI:** 10.3390/jimaging7080134

**Published:** 2021-08-05

**Authors:** Miki Tanaka, Sayaka Shiota, Hitoshi Kiya

**Affiliations:** Department of Computer Science, Tokyo Metropolitan University, 6-6 Asahigaoka, Tokyo 191-0065, Japan; tanaka-miki@ed.tmu.ac.jp (M.T.); sayaka@tmu.ac.jp (S.S.)

**Keywords:** fake images, GAN, robust hashing, tamper detection, synthetic media

## Abstract

SNS providers are known to carry out the recompression and resizing of uploaded images, but most conventional methods for detecting fake images/tampered images are not robust enough against such operations. In this paper, we propose a novel method for detecting fake images, including distortion caused by image operations such as image compression and resizing. We select a robust hashing method, which retrieves images similar to a query image, for fake-image/tampered-image detection, and hash values extracted from both reference and query images are used to robustly detect fake-images for the first time. If there is an original hash code from a reference image for comparison, the proposed method can more robustly detect fake images than conventional methods. One of the practical applications of this method is to monitor images, including synthetic ones sold by a company. In experiments, the proposed fake-image detection is demonstrated to outperform state-of-the-art methods under the use of various datasets including fake images generated with GANs.

## 1. Introduction

Recent rapid advances in image manipulation tools and deep image synthesis techniques have made generating fake images easy. In addition, with the spread of social networking services (SNS), the existence of fake images has become a major threat to the credibility of the international community. Accordingly, detecting fake/tampered images has become an urgent issue [1].

Most methods for detecting forgery assume that images are generated using specific manipulation techniques, so these methods detect unique features caused by such techniques such as checkerboard artifacts [2,3,4,5]. In contrast, tampered images are usually uploaded to social networks to share with other people. SNS providers are known to apply uploaded images to resizing and compressing [6], and such operation by an SNS provider or malicious person damage can damage or cause the images to lose the unique features of tampered images. Accordingly, methods for detecting fake/tampered images suffer from the influence of operation in general. However, conventional methods are not robust enough yet against the various types of content-preserving transforms without malice, such as resizing and compression.

**Figure 1:**
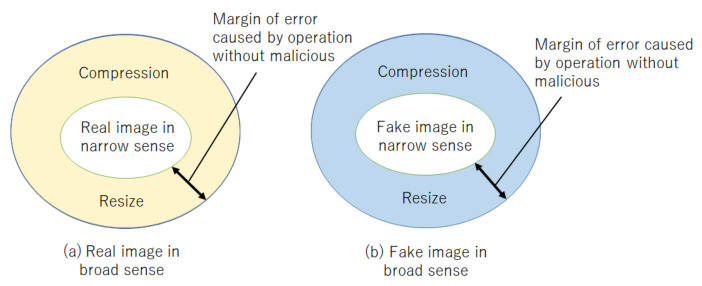
Real and fake images. (**a**) Real image in broad sense; (**b**) Fake image in broad sense.

Accordingly, the motivation of this work is to propose a novel method for robustly detecting fake/tampered images even when various operations such as compressing and resizing are applied to query images. Figure 1 illustrates two types of real and fake images, those in the narrow sense and in the broad sense, where images in the broad sense include the influence of some noise caused by resizing and image compression. The proposed method aims to robustly detect fake images even under the use of such image operations, which is well known to be applied by most SNS providers. In this paper, reference images correspond to real images in the narrow sense, so a query image is judged to be fake if it is not a real image in the broad sense.

In this paper, we investigate the possibility of developing a fake-image detection method with robust hashing for image retrieval. Hash values for fake-image detection are required to be robust enough against a number of types of image operation such as image compression and resizing. In contrast, hash values generated by using a robust hash method have to be sensitive to the influence of manipulations used for generating fake images such as copy-move and generative adversarial networks (GANs). Because of these requirements, we selected Li et al.’s method [7], and it is applied to the proposed detection method. The proposed method is a one class training method as defined by Verdoliva [1]. If there is no original hash code for comparison, the proposed method is not available for fake-image detection. In contrast, if there is an original hash code for comparison, it can more robustly detect fake images than conventional methods. In addition, the proposed method allows us to use any images, including ones generated with CNNs, as references (real images), which other detection methods, such as noiseprint and CNN-based ones, cannot be applied to.

One of the practical applications of this method is to monitor images, including synthetic ones, sold by a company. Recently, companies that sell media, including synthetic ones, have been spread [8,9,10,11], so customers can easily obtain copyright-free images suitable for the purpose of their use. However, in many cases, the manipulation of the images is limited by an acceptable use policy. The proposed method allows the companies to monitor whether the use of images is subjected to the acceptable use policy because an original hash code for comparison can be prepared, and the proposed method can be applied to any images, including synthetic images. Accordingly, the paper is posted as a detection of the unauthorized manipulation of existing images, including synthetic ones.

In an experiment, the proposed method with Li et al.’s method is demonstrated to outperform state-of-the-art methods under the use of various datasets, including fake images generated with GANs.

## 2. Related Work

### 2.1. Fake-Image Generation

Some fake images are manually generated by using image editing tools such as Photoshop. Splicing, copy-move, and deletion are carried out with such a tool. Similarly, resizing, rotating, blurring, and changing the color of an image can be manually carried out.

**Figure 2:**
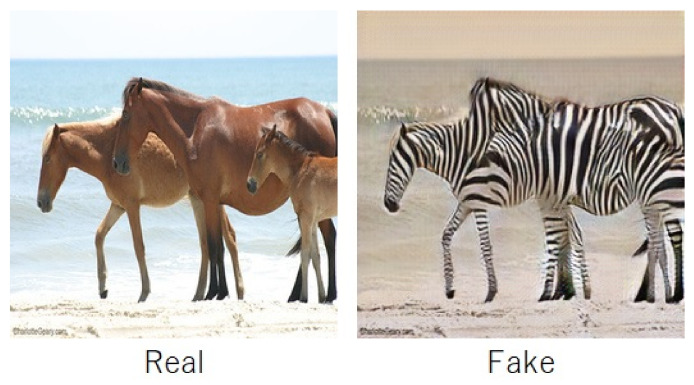
An example of a fake image in the CycleGAN dataset.

In addition, recent rapid advances in deep image synthesis techniques have made it possible to automatically generate fake images. CycleGAN [12] and StarGAN [13] are typical image synthesis techniques with GANs. CycleGAN is a GAN that performs a one-to-one transformation, e.g., changing an apple to an orange, while StarGAN is a GAN that performs a many-to-many transformation, such as changing a person’s facial expression or hair color (see Figure 2). Furthermore, fake videos created by using deep learning are called deepfakes, and various tampering methods have emerged, such as those using autoencoders, Face2Face [14], and FaceSwap [15].

Real-world fake images can include alterations by a number of operation techniques such as both image compression and resizing at the same time, even if the images are generated by using GANs. Therefore, we have to consider this when detecting real-world fake images.

### 2.2. Fake-Image Detection Methods

Image tampering has a longer history than that of deep learning. Fragile watermarking [16], the detection of double JPEG compression with a statistical method [17,18], and the use of photo-response non-uniformity (PRNU) patterns for each camera [19,20] have been proposed to detect tampering. However, these conventional methods cannot detect differences between fake images and those that have only been operated, such as resized images, without malicious intent. In addition, most of these methods do not consider detecting fake images generated with GANs.

With the development of deep learning, various fake-image detection methods with deep learning have been developed so far. These methods also assume that fake images have unique features caused by using a manipulation tool. There are several methods that use deep learning to detect fake images generated with an image editing tool, such as Photoshop. Some of them focus on detecting the boundary between tampered regions and the original image [21,22,23].

Most detection methods with deep learning have been proposed to detect fake images generated by using GANs. An image classifier trained only with ProGAN was shown to be effective in detecting images generated by other GAN models [24]. Various studies have focused on detecting checkerboard artifacts caused in two processes: forward propagation of upsampling layers and backpropagation of convolutional layers [25]. In reference [25], the spectrum of images is used as an input image in order to capture checkerboard artifacts.

To detect deepfake videos, a number of detection methods have been investigated. Some attempt to detect failures that occur in the generation of fake videos such as on the basis of poorly generated eyes and teeth [26], the frequency of blinking used as a feature [27], and the correctness of facial landmarks [28] or head posture [29]. However, all of these methods have been pointed out to have problems in terms of robustness against the difference between training datasets and test data [1]. In addition, conventional methods are not robust against images with combinations of multiple types of manipulation, i.e., resizing and deepfakes.

## 3. Proposed Method with Robust Hashing

### 3.1. Overview

**Figure 3:**
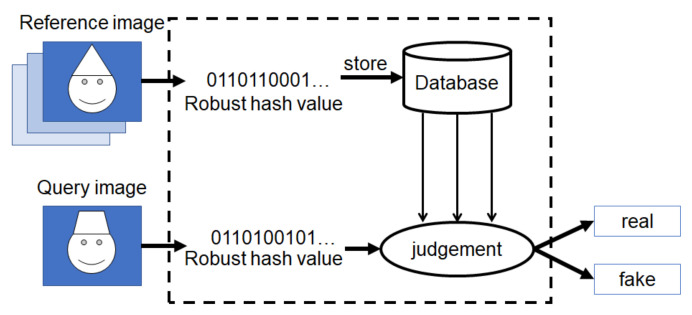
An overview of the proposed method.

Figure 3 shows an overview of the proposed method. In this framework, robust hash values are calculated from reference images by using a robust hashing method, and they are stored in a database. Similar to reference images, a robust hash value is calculated from a query image by using the hash method. The hash value of the query is compared with those stored in the database, and the query image is judged to be either real or fake in accordance with the distance between hash values.

### 3.2. Selection of Robust Hashing Methods

Hash values for fake-image detection are required to be robust enough against a number of types of image operation such as image compression and resizing since such operation does not convert the content of images, although the quality of the images is reduced. Therefore, we focus on using a robust hash method that aims to robustly retrieve images similar to query images. In contrast, hash values generated by using a robust hash method have to be sensitive to the influence of manipulation used for generating fake images such as copy-move and GANs.

Under these requirements, various robust hashing methods [7,30,31,32] are compared in terms of sensitivity and robustness in Section 4.3, in which Li et al.’s method [7] is demonstrated to have a suitable performance for fake-image detection. As mentioned above, we selected Li et al.’s method for fake-image detection.

### 3.3. Fake Detection with Robust Hashing

Li et al.’s method was designed to robustly retrieve similar images from many images with different sizes even when including noise. It has the following properties.

(a) Resizing images to 128 × 128 pixels prior to feature extraction.
(b) Performing 5 × 5-Gaussian low-pass filtering with a standard deviation of a value of one.
(c) Using features related to spatial and chromatic characteristics from images.
(d) Outputting a bit string with a length of 120 bits as a hash value.

Property (a) enhances robustness against image resizing, and property (b) can reduce the influence of image compression, respectively. Property (c) makes hash values sensitive to the influence of malicious tampering, and property (d) allows us to efficiently compare the features of reference images.

In the method, the similarity between two images is evaluated in accordance with the Hamming distance between the hash string of a query image and that of each image in a database. Let vectors $u=\{u_{1},u_{2},\cdots ,u_{n}\}$ and $q=\{q_{1},q_{2},\cdots ,q_{n}\}$, $u_{i},\;q_{i}\in \left\{0,1\right\}$ be the hash strings of reference image *U* and query image *Q*, respectively. The Hamming distance $d_{H}\left(u,q\right)$ between *U* and *Q* is given by:

(1) $$\begin{array}{c} d_{H}\left(u,q\right)≜\sum_{i=1}^{n}\delta \left(u_{i},q_{i}\right), \end{array}$$

where

(2) $$\begin{array}{c} \delta \left(u_{i},q_{i}\right)=\left\{\begin{array}{c} 0,\;u_{i}=q_{i}\\ 1,\;u_{i}\neq q_{i} \end{array}\right.. \end{array}$$

In this paper, to apply this similarity to fake-image detection, the threshold value *d* is introduced as follows.

(3) $$\begin{array}{c} \left\{\begin{array}{c} Q\in U^{\prime },\min_{u\neq q,u\in U}\left(d_{H}\left(u,q\right)\right)<d\\ Q\notin U^{\prime },\min_{u\neq q,u\in U}\left(d_{H}\left(u,q\right)\right)\geq d, \end{array}\right. \end{array}$$

where U is a set of reference images, i.e., a set of real images in the narrow sense, and $U^{\prime }$ is the corresponding set of real images in the broad sense, which does not include fake images. *Q* is judged to be either a fake image or not in accordance with Equation (3).

Note that real images are not limited to images captured by a camera under the proposed framework. Therefore, images generated by using GANs without malice can be used as reference (real) ones.

## 4. Results of Experiment

The proposed fake-image detection method with robust hashing was experimentally evaluated in terms of accuracy and robustness against various types of image operation.

### 4.1. Experiment Setup

**Figure 4:**
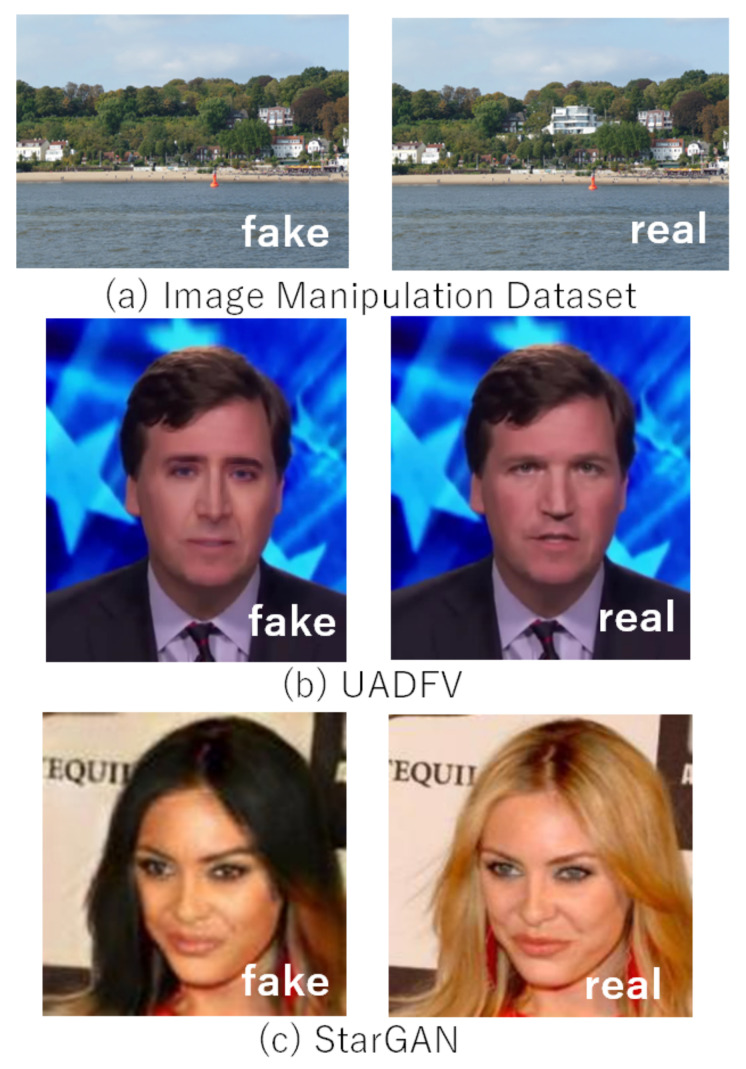
Examples of fake-images in dataset. (**a**) Image Manipulation Dataset; (**b**) UADFV; (**c**) StarGAN.

In the experiment, four fake-image datasets—Image Manipulation Dataset [33], UADFV [27], CycleGAN [12], and StarGAN [13]—were used, where each dataset consisted of pairs of a fake-image and corresponding original one (real one) as shown in Table 1 (see Figure 2 and Figure 4). For example, in the Image Manipulation Dataset, there are 48 real and 48 fake images, so the dataset consists of 96 images in total. Fake images in the Image Manipulation Dataset were generated by using image manipulation tools without GANs, and those in the other datasets were prepared with GANs. Original images were used as reference ones, where a separate reference dataset created for each experiment with a different fake-image dataset was prepared. In contrast, both original images and fake ones were used as query images, where JPEG compression with a quantization parameter of $Q_{J}=80$ was applied to all query images, so real images used as query ones included some compression noise.

The performance of fake-image detection was evaluated using two assessment criteria: average precision (AP) and F-score. AP is computed by summarizing a precision-recall curve as the weighted mean of precisions achieved at each threshold:

(4) $$\begin{array}{c} \mathrm{AP}=\Sigma_{j}\left(R_{j}-R_{j-1}\right)P_{j}\;, \end{array}$$

where $P_{j}$ and $R_{j}$ are the precision and recall at the $j_{th}$ threshold. F-score is also given by

(5) $$\begin{array}{c} F-\mathrm{score}=\frac{2RP}{R+P,} \end{array}$$

where *P* and *R* are the precision and recall at a selected threshold.

### 4.2. Selection of Threshold Value *d*

**Figure 5:**
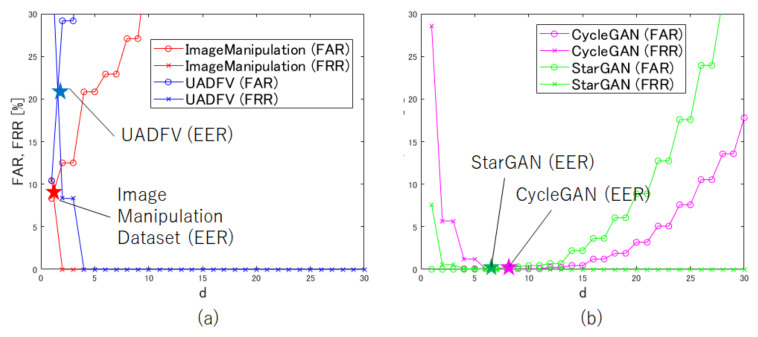
The selection of *d*. (**a**) Image Manipulation Dataset and UADFV; (**b**) CycleGAN and StarGAN.

An experiment was carried out to select threshold value *d* in Equation (3). In the experiment, equal error rate (EER) values were calculated on the datasets in Table 1, where the EER is the intersection of both the false acceptance rate (FAR) and false rejection rate (FRR) lines. Figure 5a shows experiment results under Image manipulation and UADFV, and Figure 5b shows results under CycleGAN and StarGAN. Manipulated areas in an image for CycleGAN and StarGAN were larger than those for Image manipulation and UADFV, so EER values for CycleGAN and StarGAN were given under the use of a larger threshold value than for Image manipulation and UADFV. In the experiment, d=3 was selected as a threshold value in accordance with the EER performance in Figure 5a, although the best threshold value depended on datasets.

### 4.3. Robust Hashing

Hash values for fake-image detection are required to be robust enough against a number of types of image operation, such as image compression. In addition, hash values generated by using a robust hash method have to be sensitive to the influence of manipulations used for generating fake images, such as copy-move and GANs. From these requirements, we selected four possible robust hash methods [7,30,31,32] that are robust against image compression.

To compare the four methods, they were applied to the framework of the proposed method under two datasets: the Image Manipulation Dataset and UADFV. As shown in Table 2, Li et al.’s method was confirmed to have the highest F-score among the four methods. This means that Li et al.’s method was more sensitive to the influence of manipulation used for generating fake images than the others. Accordingly, we selected Li et al.’s method for fake detection in this paper.

### 4.4. Suitability of Li et al.’s Method

**Figure 6:**
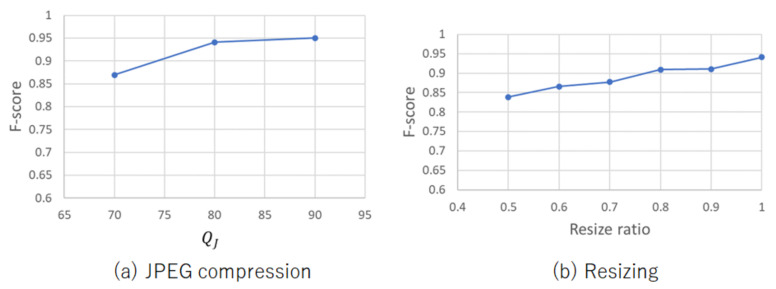
Robustness against compression and resizing (Image Manipulation Dataset). (**a**) JPEG compression; (**b**) Resizing.

A brief analysis on the suitability of Li et al.’s method to serve as the hash function was presented in Section 3.3. The analysis is verified empirically here. Figure 6 shows F-score values on the Image Manipulation Dataset under various $Q_{J}$ values and resizing rations. From the figure, the proposed method with Li et al.’s hashing was demonstrated to still maintain a high F-score value even when such image operations were applied to images. Properties (a) and (b) in Section 3.3 made a contribution to the robustness against these operations.

In Table 3, the importance of color information for fake-image detection was verified by using two image datasets, where the first dataset referred to as RGB was the Image Manipulation Dataset, and the second one called grayscale consisted of grayscale images generated from the Image Manipulation Dataset. From the table, the use of color information was confirmed to achieve a higher performance than the use of grayscale images. For Li et al.’s method, Chroma is used as a feature of images, so hash values generated by Li et al.’s method are sensitive to the change of colors. Property (c) in Section 3.3 made a contribution to the sensitivity enhancement for fake-image detection.

### 4.5. Results without Additional Operation

In this experiment, the proposed method was compared with two state-of-the-art fake detection methods, Wang’s method [24] and Xu’s method [25], where query images from the datasets were directly used without any additional operation, although real query images were compressed by using JPEG compression with $Q_{J}=80$. Wang’s method was proposed for detecting images generated by various convolutional neural networks (CNNs), including GAN models, where the classifier was trained by using ProGAN.

Table 4 shows that the proposed method had a higher accuracy than the other methods in terms of almost all criteria. In addition, the accuracy of the conventional methods heavily decreased when using the Image Manipulation and UADFV datasets. The reason is that the conventional ones focus on detecting fake images generated by using CNNs. The Image Manipulation Dataset does not consist of images generated with GANs. In addition, although UADFV consists of deepfake videos, videos in the dataset already have the influence of video compression.

### 4.6. Results with Additional Operation

Next, JPEG compression with $Q_{J}=70$ and resizing with a scale factor of 0.5 were applied to all query images in the CycleGAN dataset. Copy-move or splicing was also applied to the fake query images. Therefore, if copy-move or splicing was applied to fake queries, manipulated fake queries include the influence of two types of manipulation at the same time, such as copy-move and CycleGAN or splicing and CycleGAN.

Table 5 shows experimental results obtained with the additional manipulation, where 50 fake images generated using CycleGAN were used. The proposed method with d=7 was confirmed to still maintain a high accuracy even under the additional operation. In contrast, Wang’s method suffered from the influence of the addition operation. In particular, for splicing and resizing, Wang’s method was affected by these operations. The reason is that the method assumes that fake images are generated using CNNs to detect unique features caused by CNNs. However, splicing and resizing are carried out without CNNs. In addition, both the conventional methods had a low performance under the use of Image Manipulation Dataset and UADFV, as demonstrated in Table 4.

### 4.7. Comparison with Noiseprint Algorithm

The proposed method is a one-class training method [1], so the noiseprint algorithm [34], which is also a one-class training method, was evaluated in terms of robustness against JPEG compression and resizing.

**Figure 7:**
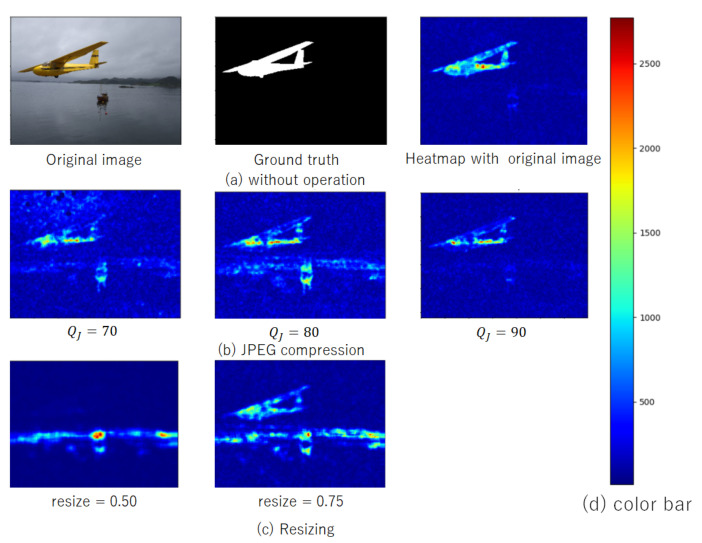
The robustness of the noiseprint algorithm against compression and resizing. (**a**) Without operation; (**b**) JPEG compression; (**c**) Resizing; (**d**) Color bar.

Figure 7 shows an example of experiment results, where an original image (fake one) with a size of 3488 × 2616, its ground truth, and the heatmap calculated from the noiseprint of the original image, as in [34], are shown in Figure 7a, respectively, and each color in the heatmap corresponds to a value, as shown in Figure 7d. From Figure 7a, it was confirmed that the noiseprint algorithm worked well under this condition. Figure 7b,c also show heatmaps calculated under the use of JPEG compression and image resizing. From these figures, the heatmaps were affected by the influence of these operations. In particular, the noiseprint algorithm failed when applying resizing with a ratio of 0.5.

**Figure 8:**
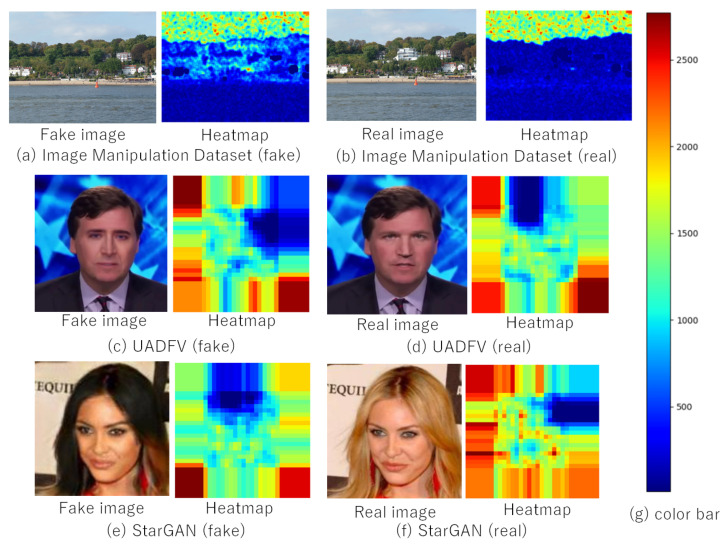
The robustness of the noiseprint algorithm no additional operation. (**a**) Image Manipulation Dataset (fake); (**b**) Image Manipulation Dataset (real); (**c**) UADFV (fake); (**d**) UADFV (real); (**e**) StarGAN (fake); (**f**) StarGAN (real); (**g**) Color bar.

Other examples are shown in Figure 8, where real and fake images were selected from three datasets: Image Manipulation, UADFV, and StarGAN, and the images had a size of 3264 × 2448, 256 × 256, and 256 × 56, respectively. Although any additional operations were not applied to the images, the noiseprint algorithm did not work well for fake-image detection. One of the reasons that images used for this experiment do not have a larger size than that of Figure 7. As described in [34], sometimes, the noiseprint algorithm does not work well under some conditions, such as the use of images with a small size and dark images. In addition, a number of attack methods against the noiseprint algorithm were proposed [35,36]. In contrast, the proposed method successfully detected the fake images shown in Figure 7 and Figure 8.

From the results shown in Table 5 and Figure 7, the state-of-the-art fake image detections and the noiseprint algorithm are not robust enough against the various types of content-preserving transformations with malice, such as resizing and compression, although they have different applications from the proposed method. From the comparison, the method was demonstrated to outperform the conventional methods in some applications in which original hash code of a reference image was prepared for comparison.

### 4.8. Computational Complexity

To evaluate the computational complexity of the proposed method, we measured the executing time of the proposed one. The Image Manipulation Dataset [33] was utilized for the evaluation. The simulation was run on a PC, with a 3.6 GHz processor and the main memory of 16 Gbytes (see Table 6). In the simulation, tic and toc function in MATLAB was used for measuring the executing time.

The executing time for computing 48 hash values from 48 queries was 2.25 s in total. In contrast, the executing time for comparing 96 hash values from query images with 48 hash values from reference images was 0.04 s in total. Li et al.’s method outputs a bit string with a length of 120 bits as a hash value, so the comparison between hash values is efficiently carried out.

## 5. Conclusions

In this paper, we proposed a novel fake-image detection method with robust hashing for the first time. Although various robust hashing methods have been proposed to retrieve images similar to the tested one, the robust hashing method proposed by Li et al. was selected for fake-image detection, considering both strong robustness against image compression and resizing and high sensitivity to the influence of manipulations used for generating fake images. When there is an original hash code for compression, the proposed method can more robustly detect fake/tampered images than conventional methods. In an experiment, the proposed method was demonstrated not only to outperform the state-of-the-art methods but also to work well even when combining multiple operations.

## Figures and Tables

**Table 1:** Datasets used in the experiment.

Dataset	Fake-Image Generation	Real	Fake
		No. of Images	
Image	copy-move	48	48
Manipulation			
Dataset [33]			
UADFV [27]	face swap	49	49
CycleGAN [12]	GAN	1320	1320
StarGAN [13]	GAN	1999	1999

**Table 2:** Comparison of robust hashing methods (F-score).

Robust Hash Dataset	Li et al.’s Method [7]	Modified Li’s Method [31]	Gong’s Method [30]	Iida’s Method [32]
Image	0.9412	0.8348	0.7500	0.768
Manipulation				
Dataset				
UADFV	0.8302	0.6815	0.6906	0.7934

**Table 3:** The influence of color information (Image Manipulation Dataset).

Dataset	F-Score
Greyscale	0.7869
RGB	0.9412

**Table 4:** Comparison with state-of-the-art methods.

Dataset	Wang’s Method [24]		Xu’s Method [25]		Proposed	
	AP	F-Score	AP	F-Score	AP	F-Score
Image Manipulation Dataset	0.5185	0.0000	0.5035	0.5192	0.9760	0.9412
UADFV	0.5707	0.0000	0.5105	0.6140	0.8801	0.8302
CycleGAN	0.9768	0.7405	0.8752	0.7826	1.0000	0.9708
StarGAN	0.9594	0.7418	0.4985	0.6269	1.0000	0.9973

**Table 5:** Comparison with state-of-the-art method under additional operation (dataset: CycleGAN).

Additional Operation	Wang’s Method [24]		Xu’s Method [25]		Proposed	
	AP	F-Score	AP	F-Score	AP	F-Score
None	0.9833	0.7654	0.9941	0.8801	0.9941	0.9800
JPEG ($Q_{J}=70$)	0.9670	0.7407	0.8572	0.7040	0.9922	0.8667
resize (0.5)	0.8264	0.3871	0.5637	0.6666	0.9793	0.5217
copy-move	0.9781	0.6400	0.9798	0.8764	1.0000	1.0000
splicing	0.9666	0.6923	0.9801	0.8666	0.9992	1.0000

**Table 6:** Machine spec used for evaluating executing time.

Processor	Intel Core i7-7700 3.60 GHz
Memory	16 GB
OS	Windows 10 Education Insider Preview
Software	MATLAB R2020a

## Data Availability

The data presented in this study are available on request from the corresponding author.
